# Japanese family physicians' attitudes, difficulties, and perceived significance in managing mental health problems: A qualitative study

**DOI:** 10.1002/jgf2.731

**Published:** 2024-09-16

**Authors:** Natsuki Kajikawa, Shoji Yokoya, Sachiko Ozone

**Affiliations:** ^1^ Department of Family Medicine, General Practice and Community Health, Faculty of Medicine University of Tsukuba Tsukuba Ibaraki Japan; ^2^ Kitaibaraki Center for Family Medicine Kitaibaraki Ibaraki Japan

**Keywords:** family physician, mental health, psychological problem

## Abstract

**Background:**

Family physicians (FPs) are expected to identify, diagnose, and treat mental health problems. Despite challenges such as limited time and low confidence in their skill as mental health providers, FPs generally consider mental health as a meaningful part of their profession. However, the role of the FP in mental health services is not clearly established in Japan. Studies have not been performed in countries without an established role of FPs' in mental health care. This study investigated Japanese FP's attitudes, difficulties, and perceived significance in managing mental health problems.

**Methods:**

Semi‐structured interviews were conducted between September 2022 and February 2023. Participants were Japanese FPs involved in mental health care. Interview transcripts were subjected to thematic analysis.

**Results:**

Thirteen FPs were interviewed. The FPs approached mental health problems “as a FP who provides holistic and comprehensive care” and “practice based on experience and self‐reflection.” The difficulties faced by FPs included “time management problems” and “referral management problems and conflicts.” Regarding significance, FPs mentioned that “gaining a deeper understanding of humanity” is important for managing mental health problems.

**Conclusions:**

The attitude of Japanese FPs toward mental health care was based on awareness of the FP's specialty. Despite difficulties such as time and referral management issues, managing mental health problems was generally considered rewarding for professional growth.

## 1 INTRODUCTION

The World Health Organization recommends integrating mental health (MH) services into primary care because it is a viable way to address MH problems, which represent an important cause of disease burden.^1^ Primary care is an important resource used by individuals with mental health problems.^2^ In addition, MH issues can manifest as physical ailments.^3^ Family physicians (FPs) are expected to detect, diagnose, and treat MH problems.

FPs face several difficulties in delivering MH care, including the patient's reluctance to seek help, lack of training, time limitations, low confidence in their ability to provide MH care, diagnostic difficulties, poor relationships with secondary MH services, and limited MH resources.^4^, ^5^ Despite these difficulties, FPs consider the management of MH issues as a meaningful part of their role.^6^, ^7^

It has been proposed that Japanese FPs should be involved in the management of MH issues. In Japan, individuals have free access to any health care provider, and they can therefore directly access psychiatrists. However, MH specialists are underutilized by Japanese individuals with MH problems because of the stigma associated with mental illness.^8^, ^9^ Japanese patients believe that consulting a non‐psychiatric specialist is as helpful as consulting a psychiatrist regarding MH issues.^10^

Although Japanese FPs receive training in psychiatry during their residency programs, the role of the FP in MH services is not established in Japan. Japanese physicians who are not psychiatrists lack confidence in the management of depression,^11^ which may also be the case for Japanese FPs. In this study, we targeted Japanese FPs who are currently involved in managing MH problems. To increase the participation of FPs in MH practice, it is necessary to clarify the attitudes of FPs and the obstacles they face, as well as the issues that they consider meaningful about managing MH problems in Japanese patients. Most studies investigating the attitudes of FPs toward the management of MH problems have been conducted in countries in which the role of FPs in MH care is recognized and established.

The purpose of this study was to investigate the attitudes and difficulties of Japanese FPs who are currently involved in managing MH problems, as well as to clarify the perceived significance of managing MH problems for FPs. The results of this study may contribute to improving the participation of FPs in MH by identifying factors or issues that need to be resolved, not only in Japan but also in other settings in which the role of FPs in the management of MH issues is not well established.

## 2 METHODS

This qualitative interview study was conducted between September 2022 and February 2023. Our study was conducted in accordance with the Standards for Reporting Qualitative Research (SRQR) guidelines.^12^

### 2.1 Participants

This study included FP specialists certified by the Japan primary care association. Psychiatrists or psychosomatic internal medicine specialists were excluded. FPs involved in managing MH problems were recruited using a maximum variation purposeful sampling strategy. In the initial stage, four physicians of different ages who practiced in different settings were selected. All of them agreed to participate in the study and introduced one or two acquaintances who worked in different settings. These physicians were contacted to ensure the participation of individuals with a variety of characteristics such as age, setting, and training background. The interviews were conducted until no additional divergent themes emerged from three consecutive interviews.^13^

### 2.2 Interviewer

Interviews were conducted by NK and SY. Both are FPs who are actively involved in MH care. NK was trained in qualitative research after obtaining a doctoral degree. SY is an experienced qualitative researcher.

### 2.3 Interviews

Semi‐structured interviews were performed face‐to‐face or online. The interviews were conducted in Japanese and lasted 45–75 min. They were recorded with an audio recorder. There was no compensation for interviewees.

According to a previous study,^14^ the scope of MH problems in our study was defined as follows: (i) common MH problems most often managed in primary care excluding dementia; and (ii) undifferentiated low mood, stress, and/or anxiety that is subclinical or not assigned a diagnostic label. MH care was defined as follows: “the management of MH problems consists of following patients beyond the initial medical visit over time for the same or subsequent condition, including medical tests and drug prescriptions (both physical and MH drugs), side‐effect monitoring, psychotherapy, or any kind of psychosocial support”.^15^

The interview was conducted following specific guidelines (Table 1). The questions addressed basic characteristics including years after graduation from medical school, clinical practice areas, training received, and problems encountered. To determine the significance of managing MH problems, FPs were directly asked to express their opinion and what they learned from their experience. As needed, the interviewer modified the order of questions and added questions when the responses required clarification.

**TABLE 1:** Interview contents.

No	Questions
1	How did you learn about managing mental health problems?
2	What do you think about the collaboration between family physicians and mental health providers? (e.g., psychiatrists, psychosomatic medicine specialists, psychotherapists, psychiatric social workers, and other related services)
3	Do you have any difficulties in managing mental health problems? Why?
4	What do you think about managing mental health problems?
5	Are you confident in your ability to manage mental health problems?
6	Have you learned anything through managing mental health problems? If so, what did you learn?
7	What do you think the family physician needs to manage mental health problems?

### 2.4 Data analysis

Data collection and analysis were performed simultaneously throughout the study period. All interviews were transcribed by NK in Japanese. The transcripts were analyzed using thematic analysis as introduced by Braun and Clarke.^16^ First, NK generated initial codes. Next, NK and SY discussed unclear codes and themes. Then, a third author who did not participate in the interviews was joined the discussion to analyze the codes and themes from different perspectives. Finally, all authors agreed about the final themes.

## 3 RESULTS

A total of 13 FPs were invited, and all of them agreed to participate in the study. None of the participants were psychiatrists or psychosomatic internal medicine specialists. The characteristics of the physicians interviewed are shown in Table 2.

**TABLE 2:** Characteristics of the study participants.

Interviewee	Age	Sex	Years after graduation	Practice location
1	43	Male	19	Rural
2	56	Male	30	Rural
3	36	Female	12	Urban
4	46	Male	23	Urban
5	34	Male	9	Rural
6	33	Male	9	Suburban
7	41	Male	15	Suburban
8	44	Male	18	Urban
9	43	Male	19	Rural
10	44	Female	19	Urban
11	40	Male	16	Rural
12	36	Male	9	Urban
13	41	Male	16	Suburban

*Note*: The Practice locations are classified according to the size of the city where the practice is located. Practices located in large cities as defined by the Japanese Ministry of Internal Affairs and Communications were classified as “urban”. Other practices were classified as “suburban” if the population was 100,000 or more, and “rural” if they were located in a city or town with a population of 100,000 or less, based on the population of the municipality where the practice was located as of January 2023.

Five main themes emerged from the interviews. Regarding the attitude of FPs toward MH problems and their management, the themes were “involvement as a FP who provides holistic and comprehensive care” and “practice based on experience and self‐reflection.” The difficulties described included “time management problems” and “referral management problems and conflict.” FPs considered that “gaining a deeper understanding of humanity” was significant for the management of MH problems.

### 3.1 Involvement as a family physician who provides holistic and comprehensive care

This theme had three sub‐themes: utilizing the skills of the FP, diagnosis based on holistic understanding and clinical experience, and the FPs mission as a physician who provides comprehensive care.

#### 3.1.1 Utilizing the skills of the FP

FPs considered their skills as family medicine specialists as beneficial for managing MH problems. Attentive listening and an empathetic attitude, which are basic skills of FPs were considered the basis of the FPs clinical practice. For the management of MH problems, FPs need to listen to their patients and understand each patient's psychosocial background. Consequently, facing MH issues increases the FPs' awareness of their communication skills, attentive listening, and empathetic attitude.

All FPs possess the necessary skills for managing MH problems, because we always do something such as lifestyle intervention using behavioral modification. By extension, we can acquire specialized skills such as behavioral therapy and psychotherapy. We learned the essential components of these therapies during family medicine residency programs. (Participant 1)

When FPs provide MH care, they listen to their patients carefully. Providing MH care improves the FPs skills in listening to patients. (Participant 5)

#### 3.1.2 Diagnosis based on holistic understanding and clinical experience

FPs followed operational diagnostic criteria (e.g., Diagnostic and Statistical Manual of Mental Disorders, 5th edition). Initially, FPs reached a diagnosis according to established diagnostic criteria, which was not difficult. After becoming familiar with the management of MH problems, they did not always adhere to diagnostic labels and instead focused on the issues that specifically affected each patient. They reached a diagnosis through a holistic understanding of patients and their clinical experience, even if the patients did not meet operational diagnostic criteria. The FPs believed that this diagnostic process allowed them to gain a deeper understanding of their patients.

Of course, I check the necessary items using operational diagnostic criteria and then make a diagnosis. Furthermore, even though a patient says, “I am anxious!”, I may determine that the diagnosis is not anxiety disorder but depression by considering their complaints. I might form an image of each disease by putting together many elements collected through my experience with each patient. I think that meeting the diagnostic criteria is sometimes not sufficient to diagnose certain psychiatric diseases. (Participant 9)

Frankly speaking, I may not reach a diagnosis because I focus on the core psychological problems of each patient and address these issues individually. (Participant 11)

#### 3.1.3 Mission as a FP who provides comprehensive care

FPs had a sense of being on a mission in which they needed to manage all health problems in the community on their first presentation. MH problems are common health problems. FPs expressed that failure to confront MH problems would prevent them from performing their duties comprehensively. They provide comprehensive care for patients despite multimorbidity. FPs considered that these characteristics distinguished them from psychiatrists.

The FP's practice is a non‐selective practice. If a patient presents with certain complaints and asks for help, we certainly listen at first and manage these issues to the best of our ability regardless of whether the problem is physical or psychological. (Participant 10)

Psychiatrists cannot prevent mild anxiety symptoms from becoming severe anxiety. Patients rarely go to psychiatrists for mild anxiety symptoms. I think that managing these types of problems is within the scope of the FP. (Participant 4)

When I talk to psychiatrists, they often separate psychiatry from other specialties based on mind‐body dualism. From our point of view, the mind and body cannot be separated. I would consider which has more weight, the mind or the body, rather than separating body from mind. (Participant 2)

### 3.2 Practice based on experience and self‐reflection

FPs were confident in their ability to manage common MH problems such as anxiety and depression based on their training in family medicine and experience in addressing MH issues in clinical practice. In cases in which psychiatrists were not available for consultation when an FP had difficulties managing patients, questions were raised regarding the appropriateness of the FP's practice. Only in a limited number of cases, FPs were able to consult a psychiatrist who was familiar with the FP's clinical practice and could offer support. The presence of this psychiatrist increased the FP's confidence by broadening the scope of the practice.

If I had to say, there are no specialists who can be easily consulted. Of course, I can discuss a patient's diagnosis and management strategies with colleagues in my clinic… We can easily refer patients to psychiatrists through formal routes. However, it is difficult for us to obtain advice from psychiatrists, even if consulting a psychiatrist is not always necessary. (Participant 10)

We are reassured when we have a MH professional who can be easily consulted regarding the management of our patients with MH problems. Just having one person to consult with enables us to manage patients more comprehensively and widely. (Participant 5)

### 3.3 Time management problems

The management of patients with MH problems is based on attentive listening, which requires longer consultation times. Therefore, FPs reported difficulties in time management because of the need to address MH issues simultaneously with medical problems. In addition, FPs described limitations in the time to provide psychotherapy, as well as an imbalance between the time spent with patients and the revenue.

Not surprisingly, it needed increased time. My consultation style is basically attentive listening, which I cannot achieve within the limited consultation times. It is difficult to allocate enough time in every day practice at the same time, isn't it? (Participant 7)

### 3.4 Referral management problems and conflict

Referring patients to psychiatrists was associated with difficulties including whether to refer a patient, resistance from patients, and long waiting times for the first appointment after a referral was made. FPs were conflicted when a referral to psychiatry failed due to patient refusal, which required the FPs to continue managing the patient on their own.

Cases presenting with psychosis or strong suicidal ideation were generally considered indications for referral to a psychiatrist. FPs did not hesitate to refer patients who met these criteria, and psychiatrists accepted the decision willingly. However, for patients without a clear diagnosis or when a patient did not improve with first‐line treatment, there was a vague consensus among FPs regarding who should be referred for psychiatric evaluation. In many cases, FPs had difficulties identifying the cases that should be referred to a psychiatrist.

Psychiatrists accept referrals for cases that interest them, or they take it for granted that conditions such as schizophrenia or bipolar disorders should be referred. Other cases such as severe suicidal ideation, self‐harm, and the risk of harming others are also accepted. Both psychiatrists and FPs are aware of clear cases that should be managed by a professional. (Participant 1)

For patients with depression who do not respond to treatment, I usually wonder whether I should refer the patient to a psychiatrist at this point or when I should make the referral. I also hesitate in cases in which the diagnosis is ambiguous and I do not know how to explain the reason for the referral to psychiatry. (Participant 7)

FPs regarded psychiatrists as specialists who excelled in pharmacotherapy and who had different assessment skills from those of FPs. Patients were sometimes reluctant to visit psychiatrists. Patients located in areas with limited MH services were often reluctant to travel the distance necessary to see a psychiatrist. To prevent patients from discontinuing MH services and reduce the patient's reluctance, FPs sometimes told patients that returning to the FP was an option if the referral was unsatisfactory.

Patients say, “Why do I end up consulting you, even though you are kind to listen to my stories”… (I explain to the patient) “First, you try to visit a psychiatrist. Just in case, I make an appointment in one month… If you cannot go to the psychiatrist or you are not satisfied with the psychiatrist, you can come back to us”…because I do not want to leave the patient unattended, I arrange it so that they can come back to my practice. (Participant 13)

After the FP referred a patient to psychiatry, there was a long waiting time to obtain a first appointment with a psychiatrist. Even if the FP made an urgent referral, the waiting time could be several months. Referring patients to a psychiatrist was therefore considered difficult for FPs when they were unable to obtain a psychiatric consultation within the desired time frame.

For example, I refer a patient who attempted suicide considering that he is a high‐risk patient after an attempt. There is a limited number of psychiatrists in my practice area, and the psychiatrists often say that we need to wait one month for an appointment. In these cases, we are unsure regarding the next step, such as for a patient who attempted suicide and cut his wrists severely. (Participant 11)

In cases in which the patient refused the referral to psychiatry, the FP remained responsible for managing the patient. In these cases, FPs had conflicting feelings because they felt that they may not be able to provide effective care for their patients.

Even if I think that a patient should be referred to a psychiatrist, the patient sometimes does not want to see a MH professional because of unnecessary reliance on the FP. As a result, I end up managing the patient. It is difficult to make a clear distinction. (Participant 6)

### 3.5 Acquiring a comprehensive understanding of humanity

FPs believed that it was important to learn about body–mind interactions, as well as understanding different values and beliefs by managing MH problems. Furthermore, this learning could lead to new discoveries and provide a deeper understanding of the FP's own value and that of others, as well as an understanding of various psychosocial backgrounds. FPs learn through experience, which they apply to their clinical practice. The relationship between a patient and the FP, which is essential for the continuity of care can be strengthened by supporting the patient's life crisis, which is a significant part of clinical practice.

Although I interpret things in a certain way, others may not share my interpretation, and I realized that different people have different approaches to the same problem. (Participant 3)

I learn a lot by listening to various stories and narratives related to their illness. When I meet a patient in a similar situation, I can imagine the problems that the patient is experiencing. (Participant 9)

At different milestones, for example getting an advanced education, marriage, having a child, and social gatherings, people are under a lot of stress and likely to become psychologically ill. From the standpoint of FPs, it is useful to consider how to confront patients who are at a peak of tension and who do not know how to overcome a life crisis. As a FP working in a clinic, it is challenging for me to confront patients in crisis in a continuous relationship. (Participant 2)

## 4 DISCUSSION

The FPs who participated in this study believed that it was part of their mission as family medicine specialists to manage MH problems. FPs also utilized their skills in family medicine and became confident through their experiences. This is consistent with previous studies describing the confidence gained from experience in managing depression by FPs.^17^ FPs described difficulties in time management and referral decisions. They thought that managing MH problems is meaningful because it allows them to grow professionally by gaining a deeper understanding of humanity as a whole.

One important finding of the study is that FPs considered the MH practice as a positive influence on their clinical practice. FPs learned about the patients' thoughts, values, and lives by managing MH problems in their practice, which requires a comprehensive understanding of patients' point of view. They perceived this as an invaluable addition to their practice and expressed that listening to the patients' narratives often led to hypotheses regarding the patients' problems. This process may accompany a process of reflexivity, which includes applying practical knowledge based on their own experience as well as that of patients or colleagues.^18^ This learning experience motivated FPs to become involved in MH management, and sharing their experience may motivate other FP's who are not involved in MH management to become involved.

Japanese FPs gain confidence in diagnosing MH problems by understanding both the medical and contextual aspects of patients. An FP's diagnosis of depression is the process of reconciling discrepancies between biomedical understanding and the patient's contextual understanding.^19^ Experience treating depressive patients can influence the FP's professional and personal growth regarding the conceptualization of depression and consequent behaviors.^20^ In this study, FPs first used established criteria to reach a diagnosis, and then became proficient in understanding both the biomedical and contextual factors. Consistent with previous studies, FPs in this study gained confidence through experience.

The results of this study showed that referral management was one of the difficulties faced by FPs. The FPs who participated in this study described the difficulties in obtaining timely access to psychiatrists for high‐risk patients who required a consultation. Psychiatrists often do not consider emergency consultations from FPs to be true emergencies when there is a lack of mutual respect and poor communication between the two professionals.^21^ FPs who collaborate with psychiatrists are less likely to think that the waiting time is too long or the communication is inadequate.^22^ Japanese MH policy has shifted inpatient care to community‐based care in recent years.^23^ However, there is still a lack of communication between psychiatrists and FPs regarding their respective roles. In this study, we found that the confidence of FPs was enhanced through positive relationships with psychiatrists. Thus, systems that facilitate smooth communication between psychiatrists and FPs are needed to improve MH care by FPs.

Lack of consultation time is an important structural barrier.^24^ Although FPs understand that listening skills are essential in the management of depression, listening requires additional time.^25^ The FPs in this study reported that their communication style requires extended consultation times in addition to the time required for diagnosis and treatment decisions. A study reported that time does not limit MH practice when FPs have confidence in their counseling skills or in their ability to allocate consultation times.^26^ The consultation time in Japan is short,^27^, ^28^ and further investigation is needed to determine the relationship between consultation time, management skills, and patient outcomes.

This study had several limitations. The interviewees who had positive attitudes toward MH practice may have failed to express negative opinions. In addition, FPs who were reluctant to manage MH problems were not included. However, the interviewers focused on difficulties in managing MH problems during the interview and obtained sufficient data on the negative aspects of the MH practice.

## 5 CONCLUSION

The attitudes of Japanese FPs toward MH practice were based on their awareness as family medicine specialists. Despite certain difficulties such as time and referral management issues, managing MH problems was considered rewarding for professional growth. By enhancing positive relationships with psychiatrists and sharing their learning experience may motivate other FP's who are not actively involved in MH management to become involved.

## CONFLICT OF INTEREST STATEMENT

The authors declare no conflicts of interest associated with this manuscript.

## ETHICS STATEMENT

Ethics approval statement: This study was conducted in accordance with the 1975 Declaration of Helsinki. Informed consent was obtained using written forms. The ethics committee of University of Tsukuba approved this study (approval number 3381).

Patient consent statement: Written consent for publication of anonymized excerpts of transcripts was obtained from all participants.

Clinical trial registration: None.

## Data Availability

The datasets analyzed in the current study are available from the corresponding author upon reasonable request.
